# Status of programmed death-ligand 1 expression in sarcomas

**DOI:** 10.1186/s12967-018-1658-5

**Published:** 2018-11-06

**Authors:** Hyung Kyu Park, Mingi Kim, Minjung Sung, Seung Eun Lee, Yu Jin Kim, Yoon-La Choi

**Affiliations:** 1 Department of Pathology, Konkuk University Medical Center, Konkuk University School of Medicine, Seoul, South Korea; 2 Department of Health Sciences and Technology, SAIHST, Sungkyunkwan University, Seoul, South Korea; 3 Laboratory of Cancer Genomics and Molecular Pathology, Samsung Medical Center, Sungkyunkwan University School of Medicine, Irwon-ro 81, Gangnam-gu, Seoul, 06351 South Korea; 4 Department of Pathology and Translational Genomics, Samsung Medical Center, Sungkyunkwan University School of Medicine, Irwon-ro 81, Gangnam-gu, Seoul, 06351 South Korea

**Keywords:** Sarcoma, PD-L1, DDLPS, UPS, IFN-γ

## Abstract

**Background:**

Sarcomas are challenging to study because of their rarity and histomorphological complexity. PD1 and PD-L1 inhibitors showed a promising anti-tumor effect in solid tumors, where a relationship between PD-L1 expression and the objective response has been evidenced.

**Methods:**

In this study, we examined PD-L1 expression in 16 bone and soft tissue sarcoma cell lines of 11 different subtypes by means of western blot, flow cytometry and immunocytochemistry, and in 230 FFPE patient-derived tumor tissues by means of immunohistochemistry using three different antibody clones. The association between PD-L1 expression and clinicopathological features was evaluated.

**Results:**

We demonstrated that PD-L1 protein is highly expressed in pleomorphic rhabdomyosarcoma, fibrosarcoma, and dedifferentiated liposarcoma (DDLPS) cell lines. From the tissue microarray, undifferentiated pleomorphic sarcoma showed ≥ 1% immunoreactivity in 20%, 17.6%, and 16.3% of the cases with PD-L1 22C3, SP263, and SP142 antibodies, respectively. In whole sections stained with a PD-L1 22C3 antibody, DDLPS showed ≥ 1% immunoreactivity in 21.9% of the cases. In DDLPS group, cases with ≥ 1% PD-L1 expression showed statistically significantly worse recurrence-free survival (*P *= 0.027) and overall survival (*P *= 0.017) rates. Upon interferon–gamma treatment, the mRNA expression levels of PD-L1 were elevated in the HS-RMS-1, LIPO-224B, MLS1765, RH30, and RH41 cell lines.

**Conclusions:**

We found that the expression of PD-L1 in sarcoma differs depending on the histologic subtype and the PD-L1 antibody clones. These results may serve as primary data for the selection of appropriate patients when applying PD1/PD-L1 inhibitor therapy in sarcoma.

**Electronic supplementary material:**

The online version of this article (10.1186/s12967-018-1658-5) contains supplementary material, which is available to authorized users.

## Background

Sarcomas are malignant mesenchymal tumors that account for approximately 1% of adult solid cancers [1]. Sarcomas can be divided into more than 50 distinct histological subtypes, and many of these subtypes are not limited to a specific location of the body [2]. Due to its rarity and morphological variability, the clinical and pathological study of sarcomas have been limited, and the mainstay of sarcoma treatment has not changed for decades [3, 4]. Surgical resection with enough safety margins remains as the only curative therapeutic option despite its limited indication and several complications. In cases with inoperable tumors, doxorubicin and ifosfamide have been used for more than 30 years and remain the mainstay for treatments. However, these cytotoxic agents are known to provide overall response rates of only about 25% in the first-line setting and are currently used for palliative, but not curative, purposes [5]. There is still a need for new treatment methods that surpass previous therapies.

After the discovery of programmed cell death protein 1 (PD-1) in 1992, PD-1 and PD-L1 have been revealed to have a fundamental role in cancer immune surveillance [6, 7]. Anti-PD-1 therapies were approved for melanoma, non-small-cell lung cancer, and various solid tumors worldwide [8–10]. Also in cases of sarcoma, several previous studies have reported that more than 50% of sarcomas, including leiomyosarcoma, dedifferentiated liposarcoma (DDLPS), undifferentiated pleomorphic sarcoma (UPS), osteosarcoma, epithelioid sarcoma, and other sarcomas, showed PD-L1 expression in tumor cells [11–13]. However, recently the SARC028 trial report described that only 4% (3/70) of the sarcoma biopsy samples (all three were from patients with UPS) were immunopositive for PD-L1 in more than 1% of tumor cells [14]. This study also reported that 11% (9/80) of the patients with sarcomas showed an objective response, especially in patients with undifferentiated sarcomas (4/10) and liposarcomas (2/10). This result is promising but also resulted in several new questions regarding the PD-L1 immunohistochemical expression rate and its role in practice.

To gain insight into the PD-L1 expression pattern in various patients with sarcomas, we examined the PD-L1 expression using various cell lines and patient tissues including both TMA and whole sections and evaluated the association between PD-L1 expression and clinicopathological features in patients with sarcomas.

## Methods

### Patient tissue specimens

A total of 230 archival formalin-fixed paraffin-embedded (FFPE) soft tissue sarcoma tissue samples, each from a different patient, were collected at the Samsung Medical Center in Seoul, Korea. Ten myxoid liposarcomas, 33 DDLPSs, and 100 UPSs were collected as tissue microarrays (TMAs), while 87 samples were analyzed as whole section from FFPE tissue blocks and comprised 32 DDLPSs, 24 myxoid liposarcomas, and 31 osteosarcomas. This study was approved by the Institutional Review Board of Samsung Medical Center in Seoul, Korea (IRB file No. 2018-03-143). Informed consents were waived by the board.

### Cell lines, reagents, and IFN-γ treatment

Human soft tissue sarcoma cell lines were obtained from American Type Culture Collection (ATCC), Korean Cell Line Bank (KCLB), and other laboratories detailed in Additional file 1: Table S1. Each cell line was grown in appropriate culture medium (Additional file 1: Table S2) with 10% fetal bovine serum (Gibco, 16000-044) and 1% antibiotic–antimycotic 100× (Gibco, 15240-112). Cell lines were tested and validated for mycoplasma detection and human cell line authentication (STR DNA profiling) using AmpFLSTR™ Identifiler PCR Amplification Kit (Thermo Fisher Scientific, 4322288). For IFN-γ treatment, each cell line was seeded into six-well plates and treated with IFN-γ (R&D systems, 285-IF-100; 50 or 100 ng/ml) or BSA (Thermo Fisher Scientific, 23209; 50 or 100 ng/ml) as controls and incubated at 37 °C for 48 h.

### Western blot

Cells were lysed in RIPA buffer (0.1% SDS, 0.5% sodium deoxycholate, 250 Mm NaCl, 1% Triton X-100 and 50 Mm pH 8.0 Tris) containing a phosphatase inhibitor and protease inhibitor cocktail tablets (Roche) and quantified using Pierce™ BCA Protein Assay kit (Thermo Fisher Scientific, 23227) according to the manufacturer’s instructions. One hundred micrograms of total protein from cells were separated by 10% SDS-PAGE and transferred to nitrocellulose membrane (Pall Corporation), then the membranes were blocked with 5% nonfat milk in 1× TBST (Tris-buffered saline with Tween 20). Proteins were probed with the following primary antibodies: monoclonal anti-PD-L1 (Cell Signaling Technology, E1L3N, 1:3000) and anti-β-actin antibodies (Santa Cruz, sc-47778, 1:1000), and washed three times with 1× TBST. Goat anti-rabbit IgG HRP (Abcam, ab6721) and goat anti-mouse IgG HRP antibodies (Abcam, ab6789) were used as secondary antibodies. Proteins were detected using ECL western blotting substrate (Promega, W1015).

### Immunocytochemistry (ICC) and immunohistochemistry (IHC)

Cells were fixed in 95% ethanol and embedded in paraffin. Egg albumin was used for cell aggregation. For ICC and IHC, 4 μm thick sections from FFPE tissue blocks were cut using a microtome and routinely deparaffinized. The sections were incubated with 0.3% hydrogen peroxide to block endogenous peroxidase activity. Antigen retrieval was performed in 0.01 M of citrate buffer (pH 6.0) or Tris–EDTA buffer (10 mM Tris at pH 9.0, 1 mM EDTA, 0.03% Tween 20) at 95 °C. Three different PD-L1 antibodies (DAKO 22C3, 1:50; VENTANA SP142, 1:50; and VENTANA SP263, 1:50) were used for immunocytochemical and immunohistochemical staining. For SP142 IHC amplification and DAB development, the Biotin-Free Catalyzed Amplification System (DAKO, K1497) was used. For SP263 IHC amplification and DAB development, the OptiView DAB IHC Detection Kit (VENTANA, 760-700) was used according to the manufacturer’s instructions. Each slide was counterstained with hematoxylin and then mounted.

To evaluate the IHC results of tissue samples including both whole sections and TMAs, each case was separated into groups with < 1% (negative), 1–49% (low), or ≥ 50% (high) positive tumor cells. A tumor cell with membranous staining, at least weak and partial, counted as a positive tumor cell.

### Flow cytometry

Cells were washed with fluorescence-activated cell sorting (FACS) buffer (filtered 0.1% BSA in PBS) and stained with phycoerythrin (PE)-conjugated monoclonal antibody specific for PD-L1 (eBioscience, MIH1) or IgG (Miltenyi Biotec, 130-092-212). Cells were filtered using a Falcon 5 ml round bottom tube with a cell strainer snap cap (Corning, 352235). Flow cytometric analysis was performed with FACSVerse and FACSuite (BD Biosciences).

### RNA extraction, cDNA synthesis, and quantitative reverse transcription PCR (qRT-PCR)

Total RNA was isolated using RNeasy Mini Kit (Qiagen, 74106) according to the manufacturer’s instructions and quantified using Nanodrop™ 2000 spectrophotometer (Thermo Fisher Scientific, ND-2000). One microgram of total RNA was used for the synthesis of cDNA. The cDNA was synthesized using the SuperScript III First-Strand Synthesis System (Invitrogen). To determine mRNA levels of *PD*-*L1* and *STAT1*, qRT-PCR was performed using SYBR Green PCR Master Mix (Applied Biosystems, 4367659) and specific primer sets (Additional file 1: Table S3). Relative mRNA expression levels were normalized to the expression level of *CTBP1* using 2^−ΔΔCt^ (mean fold change).

### Statistical analysis

Continuous variables were tested for normality of distribution using the Kolmogorov–Smirnov test and Shapiro–Wilk test. Unpaired t-test was used for the continuous variables fitting a normal distribution. Mann–Whitney U-test was used for the continuous variables showing a skewed distribution. Categorical variables were compared using the Chi square test or Fisher’s exact test. RFS was defined as the time interval between initial resection and tumor recurrence or last follow-up. OS was defined as the time interval between the initial diagnosis and death or last follow-up. Survival analysis was performed using the Kaplan–Meier method with the log-rank test. P-values ≤ 0.05 (2-tailed) was considered statistically significant. Statistical analyses were performed using Prism v.7 (GraphPad) and SPSS version 17.0 (SPSS Inc.).

## Results

### Status of PD-L1 expression in various sarcoma cell lines

**Fig. 1:**
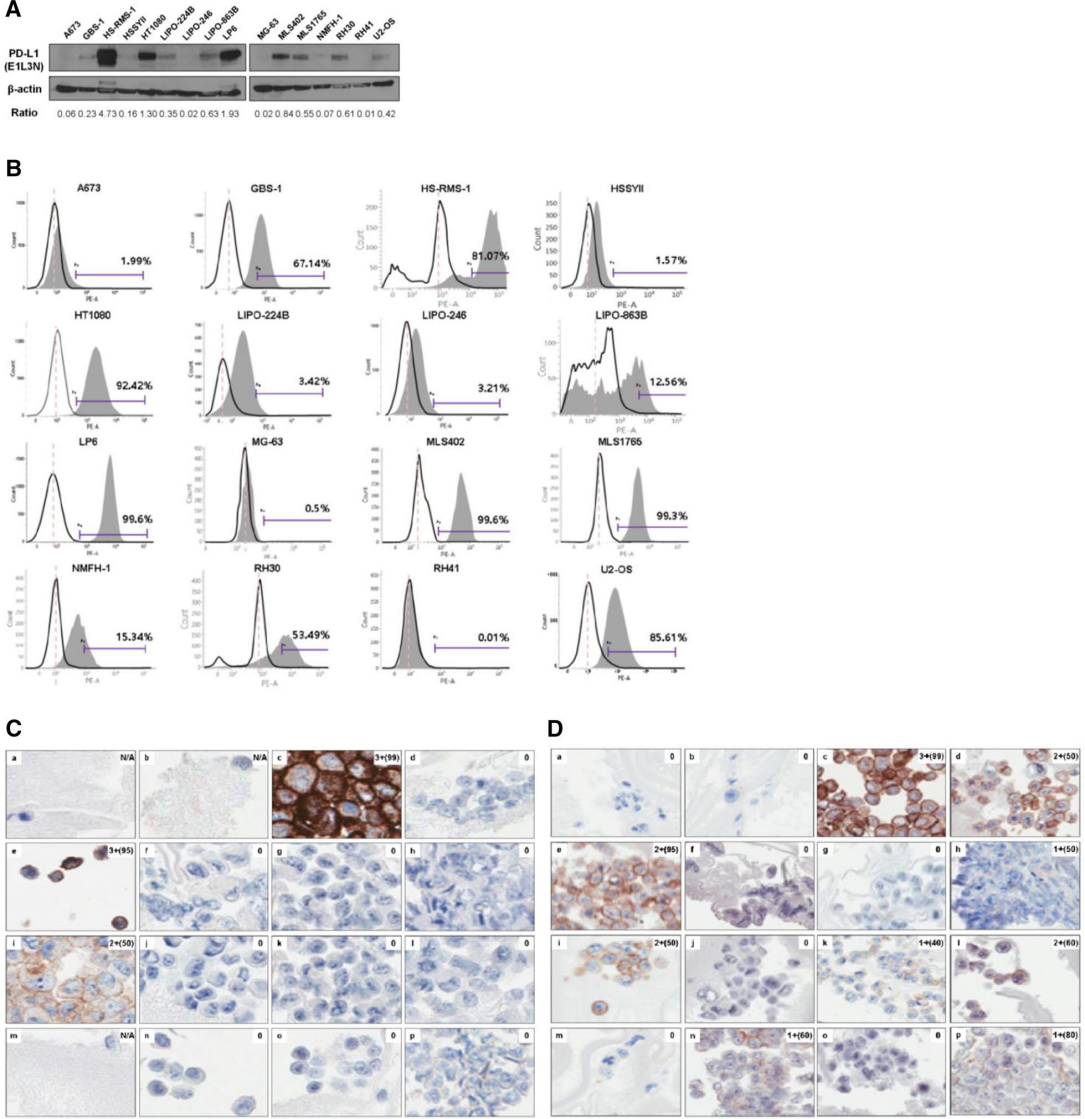
Expression levels of PD-L1 protein in various human sarcoma cell lines. **A** Total PD-L1 protein expression was determined by western blotting. The intensity of bands was quantified using ImageJ, and each band was normalized by comparing to levels of β-actin expression. **B** Cellular surface expression of PD-L1 was quantified by FACS analysis. The intensity of PD-L1 expression in human sarcoma cell lines (a–p) was measured by ICC using PD-L1 22C3 (**C**) and SP142 (**D**) antibody clones (×200 magnification). Staining intensity was graded as 0 (negative), 1+ (weak), 2+ (moderate), and 3+ (strong). The proportion of stained cells in the whole region was indicated in parallel (%). a, A673 (ewing sarcoma); b, GBS-1 (UPS); c, HS-RMS-1 (pleomorphic rhabdomyosarcoma); d, HSSYII (synovial sarcoma); e, HT1080 (fibrosarcoma); f, LIPO-224B (DDLPS); g, LIPO-246 (DDLPS); h, LIPO-863B (well-differentiated liposarcoma); i, LP6 (DDLPS); j, MG-63 (osteosarcoma); k, MLS402 (myxoid liposarcoma); l, MLS 1765 (myxoid liposarcoma); m, NMFH-1 (myxofibrosarcoma); n, RH30 (rhabdomyosarcoma); o, RH41 (rhabdomyosarcoma); p, U2-OS (osteosarcoma)

To evaluate the expression levels of total PD-L1 protein, we performed western blot on 16 human sarcoma cell lines (Fig. 1A). PD-L1 expression levels were highly elevated in the HS-RMS-1, HT1080, and LP6 cell lines, while no detectable PD-L1 expression levels were observed in the A673, LIPO-246, MG-63, NMFH-1, and RH41 cell lines. Next, to measure the expression levels of PD-L1, which is present on the cell surface, FACS was performed using the same cell lines (Fig. 1B). Consistent with the results obtained from the western blot analysis, the HS-RMS-1, HT1080, and LP6 cell lines had higher PD-L1 expression levels. Additionally, increased PD-L1 expression was found in MLS402, MLS1765, and U2-OS cell lines.

Furthermore, we prepared FFPE cell blocks with the same cell lines and then performed ICC using anti-PD-L1 antibodies (22C3 and SP142 clones) (Fig. 1C, D). To complement the fact that staining intensity of the SP142 clone has been known to be weak relative to that of the 22C3 clone, tyramide signal amplification was utilized for the IHC analysis [15, 16]. Immunostaining using the 22C3 clone demonstrated PD-L1 expression only in the HS-RMS-1, HT1080, and LP6 cell lines, consistent with the results obtained from the western blot analysis. In contrast, with the SP142 clone, PD-L1 expression was detected in 10 human sarcoma cell lines including HS-RMS-1, HT1080, and LP6.

Taken together, these data indicated that pleomorphic rhabdomyosarcoma, fibrosarcoma, and DDLPS PD-L1 expressed high level of PDL-1, as demonstrated by western blotting, FACS, and ICC results (Table 1).

**Table 1:** Summary of the expression status of PD-L1 in human sarcoma cell lines

Cell line	Origin	WB (PD-L1/β-actin ratio)	FACS (%)	ICC (22C3)	ICC (SP142)
A673	Ewing sarcoma	0.06	1.99	N/A	0
GBS-1	UPS	0.23	67.14	N/A	0
HS-RMS-1	Pleomorphic rhabdomyosarcoma	4.73	81.07	3+	3+
HSSYII	Synovial sarcoma	0.16	1.57	0	2+
HT1080	Fibrosarcoma	1.30	92.42	3+	2+
LIPO-224B	DDLPS	0.35	3.42	0	1+
LIPO-246	DDLPS	0.02	3.21	0	0
LIPO-863B	WDLPS	0.63	12.56	0	1+
LP6	DDLPS	1.93	99.6	2+	2+
MG-63	Osteosarcoma	0.02	0.5	0	0
MLS402	Myxoid liposarcoma	0.84	99.6	0	1+
MLS1765	Myxoid liposarcoma	0.55	99.3	0	2+
NMFH-1	Myxofibrosarcoma	0.07	15.34	N/A	0
RH30	Rhabdomyosarcoma	0.61	53.49	0	1+
RH41	Rhabdomyosarcoma	0.01	0.01	0	0
U2-OS	Osteosarcoma	0.42	85.61	0	1+

The staining intensity was graded as 0 (negative), 1+ (weak), 2+ (moderate), and 3+ (strong)

*UPS* undifferentiated pleomorphic sarcoma, *DDLPS* dedifferentiated liposarcoma, *WDLPS* well-differentiated liposarcoma, *N/A* not available, *WB* western blot, *FACS* fluorescence-activated cell sorting, *ICC* immunocytochemistry

### Status of PD-L1 expression in various sarcoma patient tissues

**Fig. 2:**
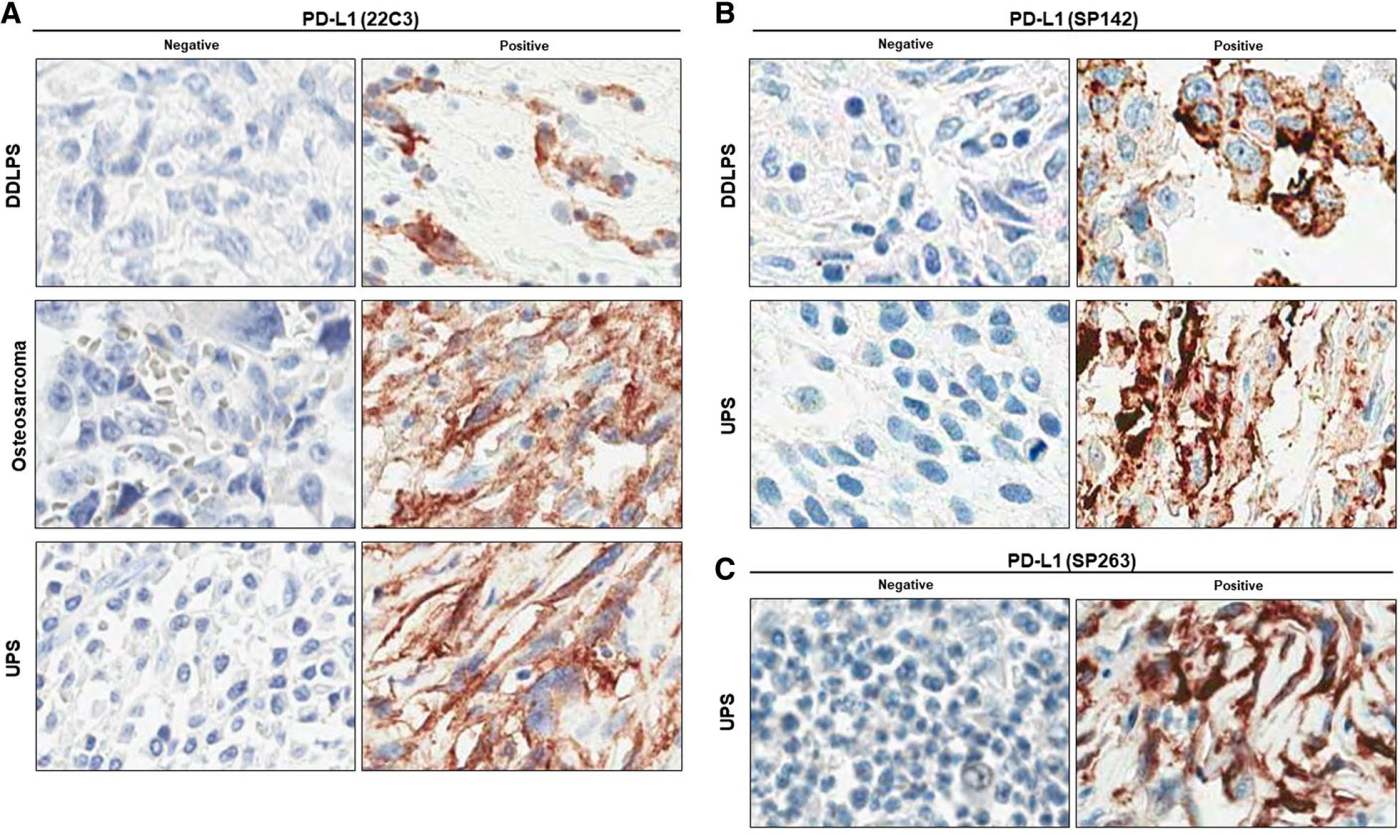
Representative cases of PD-L1 IHC in 3 sarcoma subtype tissues. Intensity of PD-L1 expression in DDLPS, osteosarcoma, and UPS was measured by IHC using PD-L1 22C3 (**A**), SP142 (**B**), and SP263 (**C**) antibody clones (×200 magnification)

Although pleomorphic rhabdomyosarcoma and fibrosarcoma cell lines showed PD-L1 immunoreactivity, these entities are rare. Therefore, we excluded them from the further evaluation using patient tissue. We included UPS and conventional osteosarcoma in consideration of their prevalence and the results of the previous studies. The results of the PD-L1 immunoreactivity evaluation are summarized in Table 2. In TMAs, no (0/28) DDLPS cases showed immunoreactivity with the PD-L1 (22C3) antibody, and one (1/29) DDLPS case showed immunoreactivity with PD-L1 (SP142) antibody in ≥ 1% of tumor cells. UPS showed ≥ 1% and ≥ 50% immunoreactivity in 20% (12/60) and 10% (6/60) of the cases, respectively, with the PD-L1 (22C3) antibody. With the PD-L1 (SP142) antibody, UPS showed ≥ 1% and ≥ 50% immunoreactivity in 16.3% (15/92) and 7.6% (7/92) of the cases, respectively. With PD-L1 (SP263) antibody, UPS showed ≥ 1% and ≥ 50% immunoreactivity in 17.6% (9/51) and 9.8% (5/51) of the cases, respectively. In whole sections stained with the PD-L1 (22C3) antibody, DDLPS showed ≥ 1% and ≥ 50% immunoreactivity in 21.9% (7/32) and 9.3% (3/32) of the cases, respectively. Finally, osteosarcomas showed ≥ 50% immunoreactivity in 3.2% (1/31) of the cases. Representative images of PD-L1-positive and -negative staining for each histologic subtype for both TMAs and whole sections are shown in Fig. 2. In a comparison of the three PD-L1 antibodies, 22C3 and SP263 showed a strong correlation (Pearson’s r = 0.882), but SP142 showed only moderate correlation with 22C3 (Pearson’s r = 0.551) and SP263 (Pearson’s r = 0.503) (Additional file 1: Table S4).

**Table 2:** Positivity on IHC in 3 subtypes of human sarcoma tissues

		22C3					SP263					SP142				
		Expression level					Expression level					Expression level				
		N/A	Negative	Low	High	Positivity	N/A	Negative	Low	High	Positivity	N/A	Negative	Low	High	Positivity
Whole	DDLPS (N = 32)		25	4	3	7/32 (21.9%)	Not tested									
	Osteosarcoma (N = 31)		30		1	1/31 (3.2%)	Not tested									
TMA	DDLPS (N = 33)	5	28			0/28 (0%)	Not tested					4	28	1		1/29 (3.4%)
	UPS (N = 100)	40	48	6	6	12/60 (20%)	49	42	4	5	9/15 (17.6%)	8	77	8	7	15/92 (16.3%)

Negative, < 1; low, ≥ 1 and < 50; high, ≥ 50. N/A and negative were not used for the calculation of the positivity rate

*DDLPS* dedifferentiated liposarcoma, *UPS* undifferentiated pleomorphic sarcoma, *N/A* not available

### Association between PD-L1 expression and clinicopathological features in DDLPS or UPS

The association between the PD-L1 expression and the clinicopathologic parameters are only available in the DDLPS whole section group (N = 32) and UPS TMA group (N = 60). The remaining groups could not be evaluated due to too their low positive rate.

**Fig. 3:**
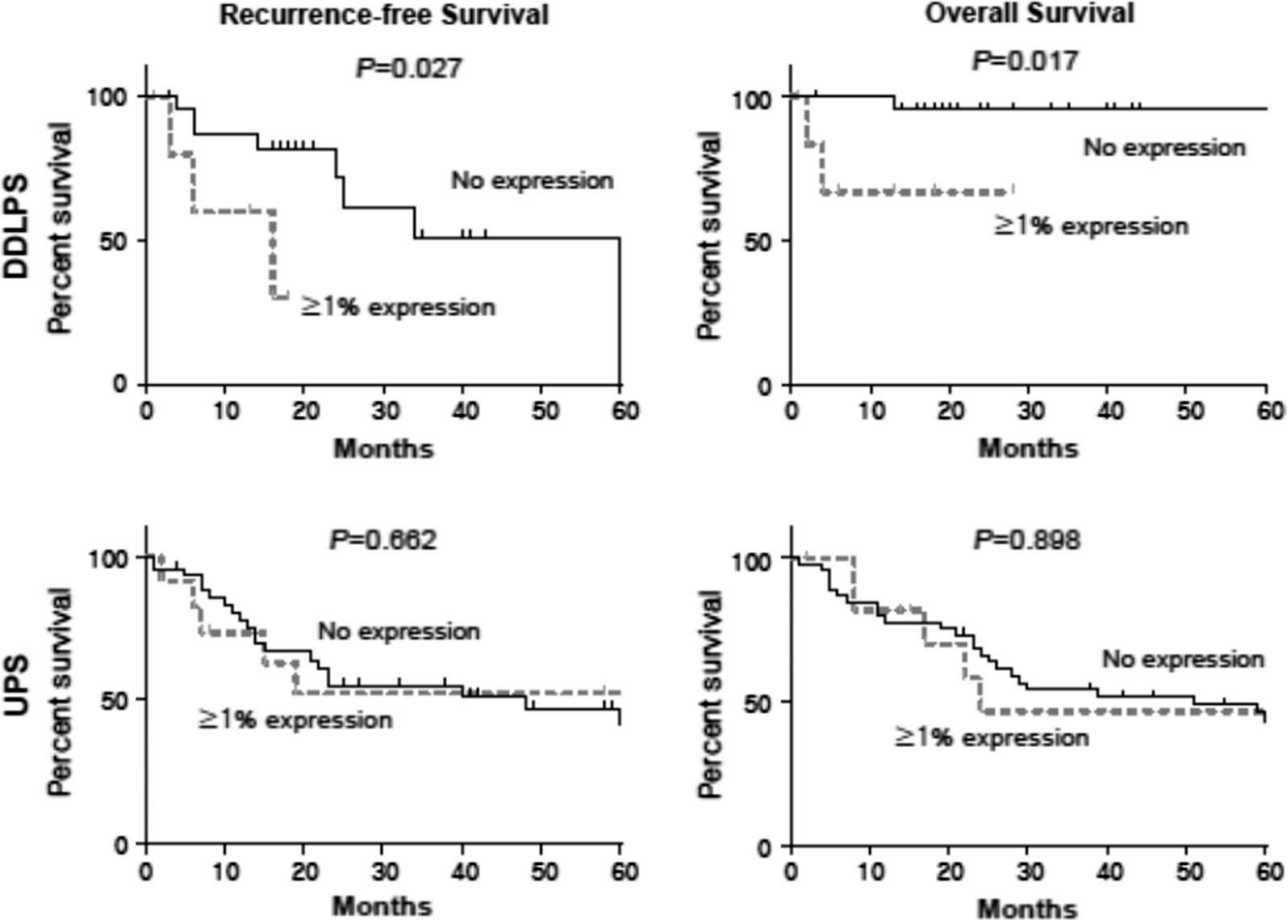
Comparison of RFS and OS between the no PD-L1 expression group and ≥ 1%PD-L1 expression group. Kaplan–Meier curves of RFS (left) and OS (right) in DDLPS (upper left and right) and UPS (lower left and right)

The clinicopathologic features of the DDLPS whole section group and UPS TMA group are summarized in Table 3. In the DDLPS group, after a median follow-up duration of 19 months, 11 patients (34.4%) had experienced recurrence, and three patients (9.4%) had died at the time of survival analysis. There were no statistically significant differences between the no PD-L1 and ≥ 1% PD-L1 expression group for clinicopathological parameters such as age, sex, tumor size, FNCLCC grade, resection margin status, and history of chemotherapy and radiation therapy. However, the RFS and OS rates were statistically significantly different between the patients with DDLPS with no PD-L1 expression and those with ≥ 1% PD-L1 expression (Fig. 3). In the UPS group, after a median follow-up duration of 49 months, 27 patients (45.0%) had experienced recurrence, and 36 patients (60.0%) had died at the time of the survival analysis. There were no statistically significant differences between the PD-L1 no expression group and ≥ 1% PD-L1 expression group for the clinicopathological parameters including RFS and OS (Fig. 3).

**Table 3:** Clinicopathologic characteristics of DDLPS (whole section) and UPS (TMA)

	DDLPS, whole section				UPS, TMA			
	Total	Negative	Positive	*P*	Total	Negative	Positive	*P*
N	32	25	7		60	48	12	
Age, year (median)	56	55	58	0.616	53	55	42.5	0.132
M:F	19:13	15:10	4:3	> 0.99	32:28	28:20	4:8	0.121
Chemotherapy, n (%)	6/32 (18.8%)	5/25 (20.0%)	1/7 (14.3%)	> 0.99	27/60 (45.0%)	21/48 (43.8%)	6/12 (50%)	0.754
Radiation therapy, n (%)	20/32 (62.5%)	15/25 (60.0%)	5/7 (71.4%)	0.683	30/60 (50%)	22/48 (45.8%)	8/12 (66.7%)	0.197
Recurrence, n (%)	11/32 (34.4%)	8/25 (32.0%)	3/7 (42.9%)		27/60 (45.0%)	22/48 (45.8%)	5/12 (41.7%)	
Expire, n (%)	3/32 (9.4%)	1/25 (4.0%)	2/7 (28.6%)		36/60 (60.0%)	29/48 (60.4%)	7/12 (58.3%)	
Tumor size (cm, median)	12.75	12.5	14	0.569	6	5.35	7	0.523
Resection margin involved, n (%)	28/32 (87.5%)	22/25 (88.0%)	6/7 (85.7%)	> 0.99	25/49 (51.0%)	21/39 (53.8%)	4/10 (40.0%)	0.496
FNCLCC								
2	28/32 (87.5%)	23/25 (92.0%)	5/7 (71.4%)	0.201	17/41 (41.5%)	14/33 (42.4%)	3/8 (37.5%)	> 0.99
3	4/32 (12.5%)	2/25 (8.0%)	2/7 (28.6%)		24/41 (58.5%)	19/33 (57.6%)	5/8 (62.5%)	

Negative, no PD-L1 immunoreactivity; positive, ≥ 1% PD-L1 immunoreactivity

*DDLPS* dedifferentiated liposarcoma, *UPS* undifferentiated liposarcoma, *RFS* recurrence free survival

### Induction of PD-L1 by IFN-γ treatment in sarcoma cell lines

Several recent studies have reported that IFN-γ induces PD-L1 expression in tumor cells by activating STAT1 signaling, suggesting a possibility on the effectiveness of immunotherapy targeting IFN-γ-induced PD-L1 expression [17–20]. Based on these findings, we examined whether IFN-γ treatment induces PD-L1 expression in sarcoma cell lines with IFN-γ. We treated the 16 sarcoma cell lines with IFN-γ, then measured the mRNA expression levels of *STAT1* and *PD*-*L1* using qRT-PCR assay.

**Fig. 4:**
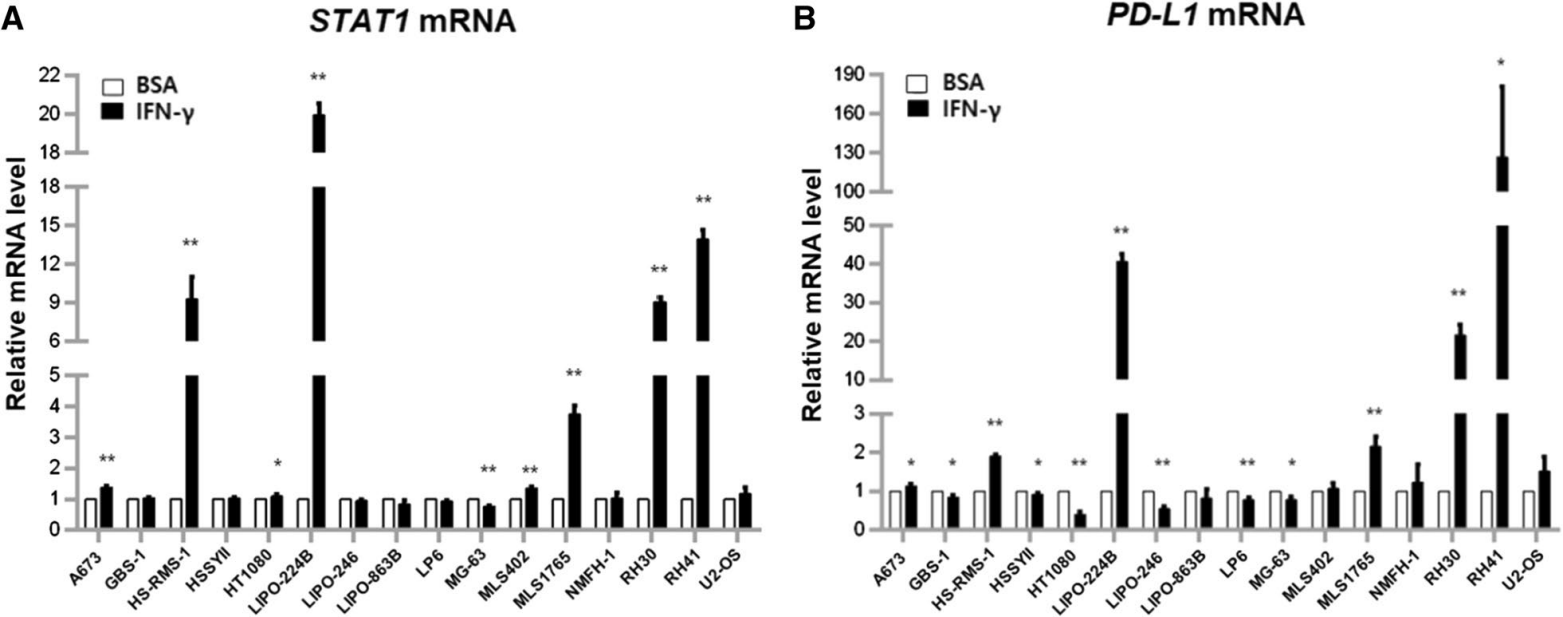
Induction of *PD*-*L1* expression by IFN-γ treatment in human sarcoma cell lines. Cells were exposed with IFN-γ (50 or 100 ng/ml) or BSA (50 or 100 ng/ml) as a control for 48 h. *STAT1* mRNA (**A**) and *PD*-*L1* mRNA (**B**) expressions were determined by qRT-PCR. Relative mRNA levels were normalized to the expression level of *CTBP1* using 2^−ΔΔCt^ (mean fold change). *P < 0.05, **P < 0.01

Upon IFN-γ treatment, the mRNA expression levels of both STAT1 and PD-L1 increased in the HS-RMS-1, LIPO-224B, MLS1765, RH30, and RH41 cell lines (Fig. 4A, B). These results suggest that *PD*-*L1* expression was induced by IFN-γ, and raises the possibility of dual therapy using IFN-γ and PD-L1 for pleomorphic rhabdomyosarcoma, DDLPS, myxoid liposarcoma, and rhabdomyosarcoma.

## Discussion

Recent advances from molecular analyses have revealed that many sarcomas are not only morphologically but also genetically distinct neoplasms. According to genomic profiles, soft tissue tumors can be broadly divided into two groups [4, 21]. The first group comprises tumors associated with specific genetic alterations and relatively simple karyotypes. Sarcoma subtypes belonging to this group include translocation-associated tumors, such as synovial sarcoma and myxoid liposarcoma, and mutation-associated tumors such as a gastrointestinal stromal tumor. Because there is a specific genomic alteration in each subtype, gene-targeted therapies are currently under investigation. On the other hand, the second group of sarcomas is those with complex karyotypes [3]. Sarcoma subtypes belong to this group includes UPS, DDLPS, pleomorphic rhabdomyosarcoma, myxofibrosarcoma, and malignant peripheral nerve sheath tumor [21, 22]. Because most of these entities have no disease-specific genomic alterations, gene-targeted therapies are of limited value. However, several recent studies have revealed that the tumors with complex karyotypes emit signals that increase their immunogenicity, and evasion of local immune surveillance plays an important role in their tumorigenesis [23]. Although the mechanisms of immune evasion are still unknown, in our opinion, the immune evasion via PD-L1 expression could be an important component.

In this study, we firstly examined the prevalence of PD-L1 expression in in a 16 sarcoma cell lines comprising 12 different subtypes. Pleomorphic rhabdomyosarcoma (HS-RMS-1), fibrosarcoma (HT1080), and DDLPS (LP6) cell lines showed a consistently increased PD-L1 protein expression via western blot, FACS, and ICC by 22C3 antibody. In a review of their genetic characteristics, both pleomorphic rhabdomyosarcoma and fibrosarcoma have been known to have complex karyotypes without specific genomic alterations [22, 24]. In cases of DDLPS, although it is defined by 12q13~15 amplifications, it is currently classified as a complex karyotype group because of additional genomic alterations during dedifferentiation [21]. In summary, cell lines that showed high PD-L1 expression all belonged to the complex karyotype group, which was consistent with our theory and a previous report [25]. On the other hand, UPS (GBS-1) and myxofibrosarcoma (NMFH-1) cell lines did not show PD-L1 expression despite their complex karyotype [21]. However, our IHC results revealed a 20% (12/60) expression rate in the TMAs of UPS. Therefore, we suggest that high PD-L1 expression tend to match with a complex karyotype group, although not all tumors with a complex karyotype express PD-L1. Screening methods such as IHC would be required in practice for the patient selection, which is similar for non-small cell carcinoma.

Although there have been several previous studies that focused on the PD-L1 immunohistochemical expression in sarcomas, most previous studies are based on a small number of specimens and showed controversial results. For example, the PD-L1 expression rates of leiomyosarcoma have been reported as 0% (0/4), 11% (1/9), and 70% (14/20) [11, 13, 26]. In this study, we used 230 sarcoma tissue samples, comprised of 87 whole sections and 143 TMAs, and three different anti-PD-L1 antibodies to solve this controversy. Our overall expression rate was 10.9% (20/184), which was lower than that of several previous studies (43–58%) [11, 13]. However, several other studies have also reported overall expression rates similar to our study (5–12%) [14, 27]. In comparison with our study and previous studies, we suggest that the overall expression rates could be related to the anti-PD-L1 antibody used for IHC. More recent studies and our study used the 22C3 clone of anti-PD-L1 antibody, which is currently used in practice, and showed similar overall expression rates. It is a well-known problem that the expression rates of PD-L1 could vary according to the antibodies used in the IHC. The Blueprint Project, which included four anti-PD-L1 antibodies used in clinical trials, showed that the staining proportion of tumor cells could vary according to the antibody clone that was used [28]. These discordances were most significant with the SP142 clone compared to the 22C3, 28-8, and SP263 clones [28]. Our results showed a strong correlation (Pearson’s r = 0.882) between 22C3 and SP263, but SP142 showed only moderate correlation with 22C3 (Pearson’s r = 0.551) and SP263 (Pearson’s r = 0.503), which was consistent with the Blueprint Project.

Histologic characteristics also can increase interobserver variability in the evaluation of PD-L1 expression of sarcomas. Under a light microscope, many sarcomas, especially UPS and DDLPS, show tumor cells intermixed with inflammatory cells. According to a recent molecular analysis report, UPS and DDLPS showed the highest median number of macrophages among sarcomas [21]. Considering that the inflammatory cells including macrophages can show PD-L1 immunoreactivity, the interpretation of PD-L1 immunoreactivity in sarcomas is not straightforward and can result in high interobserver variability. Additionally, as we described above, the recent molecular analysis revealed that many histologic sarcoma subtypes have distinct molecular characteristics. Considering that different sarcoma subtype shows different morphologies and genetics, we hypothesize that different sarcoma subtypes would show different overall PD-L1 expression rates.

DDLPS showed no PD-L1 expression in the TMAs but showed a 21.9% (7/32) PD-L1 expression rate in whole sections. This result may have been mainly due to low tumor cell proportion with PD-L1 positivity. In this study, only 3 cases showed PD-L1 expression in more than 50% of tumor cells and are usually limited to a dedifferentiated area. In a review of previous studies, there were no positive cases in the SARC028 study, which was based on biopsy samples [14]. Torabi et al. [29] reported that only one weak positive case in 64 liposarcoma cases, but they did not include DDLPS cases in their study. Osteosarcomas had a PD-L1 expression rate of 3.2% (1/31). The only case with PD-L1 immunoreactivity was conventional osteosarcoma with high-grade spindle cell morphology. Similar to our results, Torabi et al. [29] reported no (0/26) PD-L1 expression in osteosarcomas. There was also a previous study that reported that 24% (9/38) cases of osteosarcoma showed high PD-L1 RNA expression, but they did not report the immunoreactivity of PD-L1 protein in their study [30]. UPS had a 20% (12/60) PD-L1 expression rate in our study. This result was consistent with the SARC028 study which reported that only UPS cases (3/10) showed PD-L1 immunoreactivity [14]. However, both the SARC028 study and our study are based on biopsied specimens. Considering the difference in expression rates between TMAs and whole sections of DDLPS in our study, there is a possibility of underestimating the overall expression rate in UPS. In summary, in this study, we confirmed positive PD-L1 expression with the 22C3 clone in DDLPS and UPS. Our finding supports a result where pembrolizumab have a specific activity in patients with DDLPS and UPS among seven subtypes of sarcomas, which was comprised of 84 patients with bone or soft tissue sarcomas in the SARC028 study [14].

The association between PD-L1 expression and poor prognosis had been reported in many previous studies [13, 25, 30]. The cases of DDLPS in our study also showed significantly worse outcomes, whereas cases of UPS in our study showed no significant differences in outcome. Considering that UPS is a sarcoma with very poor prognosis, the contribution of PD-L1 expression to prognosis could be masked by the aggressive of the UPS.

IFN-γ has been reported to induce PD-L1 expression in several cell lines including chordoma, angiosarcoma, and osteosarcoma cell lines [17, 19, 30]. Noteworthy, PD-L1 expression was induced following vascular-targeted photodynamic treatment or ionizing radiotherapy through an increase in IFN-γ, then a mono- or combination treatment with systemic PD-1/PD-L1 pathway blockade inhibits the generation of potent local and systemic tumors in mouse models using human renal cells or murine colon cancer cells [31, 32]. These previous reports suggest the possible effectiveness of antitumor immunotherapy through a dual treatment with IFN-γ and PD-L1. We also observed the possibility of dual therapy using IFN-γ and PD-L1 in pleomorphic rhabdomyosarcoma, DDLPS, myxoid liposarcoma, and rhabdomyosarcoma. However, the effectiveness of dual therapy with IFN-γ and PD-L1 will need to be validated in various sarcoma subtypes in vivo.

## Conclusions

The encouraging result from the SARC028 study revealed the possibility of anti-PD-L1 therapy for sarcoma treatments. Precise patient selection would be mandatory for further evaluations. We showed that PD-L1 immunohistochemical reactivity could be influenced by histologic subtypes, tissue acquisition methods, PD-L1 primary antibody clone, and difficulties in interpretation. Nevertheless, anti-PD-L1 therapy in sarcomas is still largely unexplored, and further studies would allow for better patient selection and new therapeutic strategies.

## Additional files

**Additional file 1:** **Table S1.** Subtype and source of soft tissue cell lines. **Table S2.** Cell culture medium. **Table S3.** Primer sets of qRT-PCR. **Table S4.** Correlation of IHC with 3 anti-PD-L1 clones in UPS tissues (A) N = 46 (without N/A), correlation coefficient. (B) N = 100 (with N/A), correlation coefficient. (C) N = 13 (with positive expression cases in A), correlation coefficient.

## Abbreviations

PD-L1: programmed death-ligand 1
DDLPS: dedifferentiated liposarcoma
TMA: tissue microarray
UPS: undifferentiated pleomorphic sarcoma
RFS: recurrence-free survival
OS: overall survival
IFN-γ: interferon-gamma
PD-1: programmed cell death protein 1
FFPE: formalin-fixed paraffin-embedded
ATCC: American Type Culture Collection
KCLB: Korean Cell Line Bank
ICC: immunocytochemistry
IHC: immunohistochemistry
FACS: fluorescence activated cell sorting
PE: phycoerythrin
