# Supplementary material for: Status of programmed death-ligand 1 expression in sarcomas
Source: J Transl Med. 2018 Nov 6;16:303. doi: 10.1186/s12967-018-1658-5 (PMC6219031; doi:10.1186/s12967-018-1658-5)
Supplement: Supplementary file 1 — Additional file 1: Table S1. Subtype and source of soft tissue cell lines. Table S2. Cell culture medium. Table S3. Primer sets of qRT-PCR. Table S4. Correlation of IHC with 3 anti-PD-L1 clones in UPS tissues (A) N = 46 (without N/A), correlation coefficient. (B) N = 100 (with N/A), correlation coefficient. (C) N = 13 (with positive expression cases in A), correlation coefficient. [file 12967_2018_1658_MOESM1_ESM.docx]

**Table S1.** Subtype and source of soft tissue cell lines

| **Cell line** | **Sarcoma subtype** | **Source** |
| --- | --- | --- |
| A673 | Ewing sarcoma | ATCC (American Type Culture Collection) |
| GBS-1 | UPS | Dr. Hiroaki Kanda |
| HS-RMS-1 | Pleomorphic rhabdomyosarcoma | Dr. Hiroshi Sonobe |
| HSSYII | Synovial sarcoma | Dr. Hiroshi Sonobe |
| HT1080 | Fibrosarcoma | KCLB (Korean Cell Line Bank) |
| LIPO-224B | DDLPS | Dr. Dina Lev |
| LIPO-246 | DDLPS | Dr. Dina Lev |
| LIPO-863B | WDLPS | Dr. Dina Lev |
| LP6 | DDLPS | Dr. Jonathan A. Fletcher |
| MG-63 | Osteosarcoma | KCLB (Korean Cell Line Bank) |
| MLS402 | Myxoid liposarcoma | Dr. Pierre Åman |
| MLS1765 | Myxoid liposarcoma | Dr. Pierre Åman |
| NMFH-1 | Myxofibrosarcoma | Dr. Akira Ogose |
| RH30 | Rhabdomyosarcoma | Dr. Peter J. Houghton |
| RH41 | Rhabdomyosarcoma | Dr. Peter J. Houghton |
| U2-OS | Osteosarcoma | KCLB (Korean Cell Line Bank) |

UPS, undifferentiated pleomorphic sarcoma; DDLPS, dedifferentiated liposarcoma; WDLPS, well-differentiated liposarcoma.

**Table S2.** Cell culture medium

| **Cell line** | **Medium** |
| --- | --- |
| A673 | RPMI 1640 |
| GBS-1 | RPMI 1640 |
| HS-RMS-1 | RPMI 1640 |
| HSSYII | DMEM |
| HT1080 | RPMI 1640 |
| LIPO-224B | DMEM |
| LIPO-246 | DMEM |
| LIPO-863B | DMEM |
| LP6 | RPMI 1640 |
| MG-63 | MEM |
| MLS402 | RPMI 1640 |
| MLS1765 | RPMI 1640 |
| NMFH-1 | RPMI 1640 |
| RH30 | RPMI 1640 |
| RH41 | RPMI 1640 |
| U2-OS | DMEM |

**Table S3.** Primer sets of qRT-PCR

| **Primer name** | **Primer sequence** |
| --- | --- |
| CTBP1_F | GGGAGATCCGCAGAGCCAT |
| CTBP1_R | GGTCCTTGTTGACACAGTTCTTC |
| PD-L1_F | CCAAGATACAAACTCAAAGAAGCA |
| PD-L1_R | CCGATGAACCCCTAAACCAC |
| STAT1_F | GCTGAGTTGGCAGTTTTCTTCT |
| STAT1_R | ATCGGGGCTGGCGTTAGG |

**Table S4.** Correlation of IHC with 3 anti-PD-L1 clones in UPS tissues

A. N=46 (without N/A), correlation coefficient

|  | | **22C3** | **SP142** | **SP263** |
| --- | --- | --- | --- | --- |
| **22C3** | **Pearson correlation** | 1 |  |  |
|  | **p-value** |  |  |  |
| **SP142** | **Pearson correlation** | 0.537 | 1 |  |
|  | **p-value** | <0.001 |  |  |
| **SP263** | **Pearson correlation** | 0.871 | 0.497 | 1 |
|  | **p-value** | <0.001 | <0.001 |  |

B. N=100 (with N/A), correlation coefficient

|  | | **22C3** | **SP142** | **SP263** |
| --- | --- | --- | --- | --- |
| **22C3** | **Pearson correlation** | 1 |  |  |
|  | **p-value** |  |  |  |
| **SP142** | **Pearson correlation** | 0.551 | 1 |  |
|  | **p-value** | <0.001 |  |  |
| **SP263** | **Pearson correlation** | 0.882 | 0.503 | 1 |
|  | **p-value** | <0.001 | <0.001 |  |

C. N=13 (with positive expression cases in A), correlation coefficient

|  | | **22C3** | **SP142** | **SP263** |
| --- | --- | --- | --- | --- |
| **22C3** | **Pearson correlation** | 1 |  |  |
|  | **p-value** |  |  |  |
| **SP142** | **Pearson correlation** | -0.365 | 1 |  |
|  | **p-value** | 0.220 |  |  |
| **SP263** | **Pearson correlation** | 0.693 | -0.184 | 1 |
|  | **p-value** | 0.009 | 0.546 |  |
